# Weight Loss and Impact on Quality of Life in Parkinson’s Disease

**DOI:** 10.1371/journal.pone.0124541

**Published:** 2015-05-04

**Authors:** Umer Akbar, Ying He, Yunfeng Dai, Nawaz Hack, Irene Malaty, Nikolaus R. McFarland, Christopher Hess, Peter Schmidt, Samuel Wu, Michael S. Okun

**Affiliations:** 1 Department of Neurology, Brown University, Providence, Rhode Island, United States of America; 2 Department of Mathematics, Clarkson University, Potsdam, New York, United States of America; 3 Department of Biostatistics, University of Florida, Gainesville, Florida, United States of America; 4 Department of Neurology, University of Florida, Gainesville, Florida, United States of America; 5 National Parkinson Foundation, Miami, Florida, United States of America; University of Toronto, CANADA

## Abstract

**Introduction:**

Weight loss is common in Parkinson’s Disease (PD) and sometimes may precede the diagnosis. Weight loss is associated with multiple factors but its impact on health-related quality of life (HRQL) in PD remains unknown. We sought to investigate the factors associated with weight change and to quantify its effect on HRQL.

**Methods:**

The National Parkinson Foundation Quality Improvement Initiative (NPF-QII) data was used to analyze PD patients longitudinally between two visits, separated by 12±6 months. Multiple linear regression analyses were used to assess the associations between baseline covariates and body weight change per month, and to evaluate whether, and to what degree, Parkinson’s Disease Questionnaire (PDQ-39) scores were affected.

**Results:**

A higher Hoehn & Yahr stage, higher number of comorbidities, older age, lower MOCA estimate, and higher rate of levodopa usage were observed in patients who lost weight. Multivariate regression analysis indicated that age and levodopa usage were significantly associated with weight loss. Furthermore, monthly body weight loss was significantly associated with HRQL decline in PD patients. Loss of 1 lb (0.45 kg) per month was associated with a decline in QOL: an increase of 0.5% in PDQ-39 Summary Index score (p=0.004), and 1.1% and 1.5% increases in the mobility and ADL dimensions, respectively.

**Conclusion:**

Weight loss in PD is common and seems to correlate with worsened HRQL. Awareness of factors associated with weight loss and its relation to HRQL may help practitioners improve patient management and expectations.

## Introduction

Parkinson’s disease (PD) is a chronic progressive neurodegenerative syndrome that impacts both motor and non-motor domains.[1] Treatment of PD is largely symptomatic and much of the focus has been on improvement of function and health-related quality of life (HRQL).[1] PD treatment frequently targets the motor symptoms including tremor, rigidity, slowness, and gait impairment. However, there are many non-motor features that frequently emerge, including impaired sensory perception (anosmia, dysgeusia, tingling and pain), mood changes (depression, anxiety), cognitive decline (executive dysfunction, slowed thinking), dysautonomia (constipation, orthostatic hypotension, erectile dysfunction), and apathy (with or without depression).[2] These symptoms may individually, or in combination, lead to a decreased food intake or, alternatively, to an increased energy expenditure with the possibility of consequent weight loss.[3–6] In addition, the neurodegenerative process itself may affect regions of the brain important to the maintenance of weight (e.g. hypothalamus).[7]

Weight loss is a common feature of PD, and may predate expert diagnosis. Chen et al.[3] observed that in the 10 years preceding a PD diagnosis, the mean body weight decreased by 5.2 pounds (lbs) Although body weight was unchanged 2 to 4 years prior to PD diagnosis, weight decreased by 7.7 lbs over the next 8 years. Additionally, PD stage has also been correlated to weight loss.[8] The current study asks an important question about weight loss in PD, and whether it correlates with HRQL.

We sought to examine PD weight loss and its association with HRQL. Using a prospectively compiled longitudinal PD outcomes database, we quantified the effect of the mean monthly PD weight loss and analyzed the consequent impact on HRQL. Our hypothesis was that patients with more advanced disease would have lower body weight and worse HRQL. We further hypothesized that longitudinal weight loss would adversely affect HRQL.

## Methods

### Data source

The National Parkinson Foundation (NPF) prospectively compiles and maintains long-term clinical outcomes of people with PD drawn from fifteen Centers of Excellence within and outside of the United States as part of the NPF Quality Improvement Initiative clinical study (NPF-QII). Patients who consent to enrollment are annually examined in-person. The outcomes project collects demographic information, disease onset, duration, stage and symptom-severity, living conditions, comorbid conditions, medications, other treatments/referrals, and clinical condition, as well as outcomes of several validated measures including the timed up-and-go (TUG) test, several cognition measures, the Parkinson’s Disease Questionnaire [9] (PDQ-39), and multi-dimensional caregiver strain. Since the NPF-QII data is de-identified, a full review by the institutional review board was not required.

### Subject selection & definitions

The design was a retrospective cohort study using the data prospectively collected from the NPF-QII longitudinal dataset. All PD patients with two subsequent visits separated by 12 months (±6 months) were included. The variables analyzed included age, gender, age at onset, disease duration, living situation, presence of a regular care partner, Hoehn & Yahr (H & Y) stage, presence of rest tremor, motor fluctuations, TUG test, immediate and delayed word recall, verbal fluency, MOCA estimate, number of comorbidities, usage of levodopa, antidepressants, antipsychotics, speech therapy, exercise, social worker utilization, and mental health referral. HRQL was measured by a validated scale, the PDQ-39. The PDQ-39 Summary Index score (PDQ-SI), as well as its ADL and mobility dimensions, were analyzed.

As part of the data collection process, the NPF-QII instructs examining clinicians to estimate their diagnostic certainty of PD as being more than 90%, 50–90%, or less than 50%. Only subjects who were classified as having PD with greater than 90% certainty were included for analysis.

The 25^th^ percentile of absolute weight change was less than 2 lbs (0.9 kg), which was used as cutoff of “no change”. Subjects who lost ≥2 lbs (0.9 kg) were compared to subjects who lost <2 lbs or had no change. In addition, we treated patients whose weight change was more than 3 standard deviations away from the sample mean as outliers. Their body weight may have been erroneously entered, or caused by a medical comorbidity (e.g., malignancy).

Collection of data was conducted under the approval of the University of Florida IRB-01 (approval #308–2009). Patient records in the NPF-QII data were de-identified prior to analysis.

### Statistical analysis

A cross-sectional analysis was conducted for PD patients at the first visit. Baseline demographic and clinical characteristics were compared between patients who lost weight versus those who gained weight or had no change. The analysis of variance for continuous variables (ANOVA) and a chi-square test or a fisher-exact test for categorical variables were utilized. The effect of weight change on HRQL was analyzed longitudinally for patients with available data at visits 1 and 2. “Monthly weight change” was calculated by dividing the change in body weight (in pounds) by the follow-up interval (in months). For patients with available data at the follow-up visit (12±6 months), multiple linear regression analyses were used to assess the associations between baseline covariates and body weight change per month. Further, the impact of monthly weight change on HRQL measures (PDQ-SI score and ADLs and mobility dimensions) was analyzed while adjusting for covariates selected from a list of 18 pre-specified factors (listed above). Commercially available SAS software (version 9.2) was used to perform the statistical analysis. All tests were two-sided and p-values less than 0.05 were deemed statistically significant.

## Results

A total of 5443 PD patients had first visit data available at the time of the data request and 4633 patients were diagnosed with idiopathic PD with reported greater than 90% certainty by a movement disorder specialist at one of the NPF Centers of Excellence. The analyses included 1718 patients, after exclusion of those patients with no data available at a second visit (n = 2476), those whose follow-up visit date was not between 6 and 18 months from the first visit (n = 355), and 84 patients with no weight data or with a weight change greater than three standard deviations (mean 28 lb or 12.7kg).

Baseline clinical characteristics, demographic data, and social variables are presented in Table 1. A higher Hoehn & Yahn stage, higher number of comorbidities, older age, lower MOCA estimate, and higher rate of levodopa usage were observed in patients who lost weight more than 2 lbs (0.9 kg) as compared to patients who gained weight or had no change. Furthermore, based on multivariate regression analysis, we found that older age and levodopa usage were associated with larger weight loss per month. Specifically, when age was controlled, patients on levodopa lost approximately 0.18 lb (0.08 kg) per month more than patients not on levodopa (p = 0.007); and controlling for levodopa use, a ten year increase in age was associated with a mean loss of 0.08 lb (0.04 kg) per month (p = 0.001).

**Table 1:** Demographic, clinical and social variables.

		All subjects	Subjects with weight loss*	Subjects with weight gain or no change	P-value
		(n = 1718)	(n = 757)	(n = 961)	
Hoehn & Yahr stage	1–1.5	189	75 (10.3%)	114 (12.3%)	**0.013**
	2–2.5	935	402 (55.1%)	533 (57.5%)	
	3–3.4	446	222 (30.5%)	224 (24.2%)	
	4 or over 4	86	30 (4.1%)	56 (6.0%)	
Standardized TUG**		-0.18±0.95	-0.19±0.95	-0.18±0.95	0.776
Antidepressant medications used		501	226 (30.1%)	275 (28.7%)	0.553
Number of comorbidities		1.7±1.3	1.8±1.3	1.6±1.3	**0.002**
Age		66.3±9.6	67.5±9.4	65.3±9.7	**<.0001**
Disease duration		9.2±6.0	9.3±5.9	9.1±6.1	0.364
Moca estimate		24.4±3.3	24.1±3.3	24.7±3.3	**0.002**
Social worker/counseling		200	93 (12.3%)	107 (11.1%)	0.465
Presence of Motor fluctuations		805	369 (49.1%)	436 (45.6%)	0.142
Antipsychotic medications		74	37 (4.9%)	37 (3.9%)	0.285
Regular care partner	Spouse/partner	224	107 (14.2%)	117 (12.2%)	0.742
	Other relative	1392	603 (79.9%)	789 (82.2%)	
	Paid caregiver	71	31 (4.1%)	40 (4.2%)	
	Other	22	11 (1.5%)	11 (1.1%)	
	No	6	3 (0.4%)	3 (0.3%)	
Mental health treatment or referral		162	65 (8.6%)	97 (10.1%)	0.289
Speech therapy		195	92 (12.2%)	103 (10.7%)	0.360
Gender					
Male		1089	470 (62.1%)	619 (64.4%)	0.321
Female		629	287 (37.9%)	342 (35.6%)	
Levodopa usage		1450	675 (89.2%)	775 (80.7%)	**<0.0001**
Presence of rest tremor		1240	565 (75.2%)	675 (71.1%)	0.054
PDQ-Mobility		11.3±10.4	11.7±10.2	11.0±10.5	0.181
PDQ-ADL		6.9±5.5	6.9±5.4	6.9±5.6	0.963
MCSI total		16.7±15.2	17.3±15.5	16.2±15.0	0.206
Dopamine agonist		715	298 (39.4%)	417 (43.5%)	0.086

* 25% of patients had less than 2 lb absolute weight change; patients with 2lb or more decrease were designated as losing weight.

** Smaller standardized TUG means using less help or taking less time to stand up and go.

In addition to monthly body weight change, two factors were observed to be significantly associated with improvement in HRQL: referral to or treatment by a mental health specialist and presence of resting tremor. After adjusting for these two covariates, it was observed that with each 1 lb (0.45 kg) decrease in body weight per month, the change in the mean PDQ-SI increased (HRQL worsened) by 0.729 (0.5% of 156 total points, p = 0.019). No significant interaction was found between weight change and other factors (see Table 2). Similar associations were also observed between body weight change per month and the subscales of mobility and ADLs. The mean PDQ-mobility dimension and the mean PDQ-ADL dimension increased by 0.444 (1.1% of 40 total points; p = 0.077) and 0.360 (1.5% of 24 total points; p = 0.013) respectively, with each 1 lb (0.45 kg) loss in body weight per month.

**Table 2:** Regression analysis of change in PDQ-39 total score.

Variable		Full Model			Final Model		
Name	Level	Estimate	Standard Error	P-Value	Estimate	Standard Error	P-Value
Intercept		4.950	6.026	0.412	-1.013	1.089	0.353
Weight change per month		-0.769	0.364	**0.035**	-0.729	0.311	**0.019**
Hoehn & Yahr stage (reference level: 4–5)							
	1	-0.702	1.671	0.675			
	2	-0.742	1.446	0.608			
	3	-0.135	1.410	0.924			
Standardized TUG		-0.355	0.346	0.305			
Antidepressant use		-0.331	0.614	0.590			
Number of comorbidities		-0.109	0.216	0.614			
Age		0.011	0.033	0.735			
Disease duration		0.010	0.051	0.837			
MOCA estimate		-0.083	0.089	0.352			
Lack of social worker/counseling		1.357	0.844	0.108			
Presence of motor fluctuations		0.842	0.588	0.152			
Antipsychotic medications		1.888	1.351	0.163			
Regular care partner (Reference level: No)							
	Spouse/Partner	-3.786	4.596	0.410			
	Other Relative	-3.632	4.547	0.425			
	Paid Caregiver	-1.291	4.726	0.785			
	Other	-4.418	5.208	0.396			
Mental health treatment or referral		-2.078	0.958	**0.030**			
Speech therapy		0.950	0.871	0.275			
Male gender		-0.815	0.568	0.151			
Levodopa usage		-0.100	0.835	0.905			
Presence of rest tremor		-1.491	0.613	**0.015**	-1.336	0.581	**0.022**
Dopamine agonist		-0.852	0.555	0.125			

## Discussion

This study aimed to determine whether weight changes in PD patients correlate with clinical characteristics, social factors, and HRQL outcomes. The results reveal that interval weight loss is associated with a higher H&Y stage, higher number of comorbidities, older age, a lower MOCA estimate, and a higher rate of levodopa usage. We demonstrate significant correlations between interval weight loss and decline in HRQL (PDQ-SI, and mobility and ADLs dimensions).

Increasing age is one of several factors previously shown to be strongly associated with weight loss in PD.[5] Older age, worse MOCA scores and higher rates of levodopa usage are all suggestive of disease progression. In addition to the general frailty which accompanies aging, PD patients with later-onset disease progress more rapidly, and are at particular risk for becoming unable to care for themselves.[10] Furthermore, younger PD patients requiring medication are usually initiated on dopamine agonists which can precipitate compulsive eating and weight gain.[11] Our results demonstrate a trend, without statistical significance, for fewer weight-losing subjects to be on a dopamine agonist.

In addition to monthly weight loss, two other covariates negatively affected change in HRQL: lack of referral to a mental health expert, and absence of rest tremor. In our cohort, we did not observe significant correlations between other factors previously reported, such as H&Y and MOCA, which may relate to the potential bias of patients drawn from the expert clinicians. Controlling for the two covariates found (mental health referral and absent rest tremor), each 1 lb (0.45 kg) weight loss led to a statistically significant worsening of the PDQ-SI by 0.5%, mobility dimension by 1.1%, and ADL dimension by 1.5%. The practical effect of these small changes in HRQL on a monthly basis may not appear meaningful, but when taken in aggregate over a longer follow-up interval, they become significant and suggest that weight loss should be carefully monitored and addressed in PD patients. For example, the loss of 5 lbs (2.25 kg) noted during a patient’s routine follow-up visit would be associated with an estimated decline in HRQL by 2.5%, 5.5% and 7.5% in PDQ-SI, mobility and ADL scores. Using this method as a tool may possibly prompt an investigation to elucidate the cause of weight loss and discuss potential management options to improve HRQL.

PD weight loss cannot be attributed entirely to any one factor.[12, 13, 3, 5] Two review articles have been published with more thorough discussions about nutritional status and weight change in PD.[12, 13] Many elements impact food intake and energy expenditure in PD, however, these factors do not completely account for weight loss. These factors may include higher H&Y stage, increasing age, use of levodopa, worsening cognition, development of motor fluctuations, absence of tremor, and low overall physical activity.[12, 13, 3, 5, 6] Though mainly a hypokinetic movement disorder, several features of PD can potentially contribute to an increased energy expenditure, including tremor, rigidity and dyskinesia. Studies of resting energy expenditure in PD have demonstrated increased calorie consumption.[4, 14, 15] The notion that this increase in energy expenditure is the major cause of weight loss in PD is supported by the observation that weight loss precedes the diagnosis even when there is an increase in energy intake.[3, 4, 14] This view is opposed by a study demonstrating decreased energy expenditure by PD patients compared to controls.[16]

Dysfunctional smell and taste perception, apathy, depression, slowed digestion and dysphagia all may individually, or in combination, affect weight.[13, 17, 18, 6] Additionally, the motor symptoms of PD may impact weight through direct and indirect effects on shopping, cooking, feeding, and socialization.[19, 20] Medications for PD symptoms may result in nausea and can be less effective when taken with food. Furthermore, inadequate absorption of food due to gastroparesis may result in a decreased availability of energy. Finally, cognitively impaired PD patients may not attend to consuming enough calories due to forgetfulness, especially when a caregiver is absent.[18, 6]

It is unclear whether reversing weight loss in PD patients will improve HRQL. Since PD patients typically return to the physician for routine follow up every 3 to 6 months, a practical recommendation may be to closely monitor weight at each visit,[12, 21] and to discuss modifiable factors such as proper nutrition and eating habits. This study did not address whether intervention to modify weight would affect HRQL. Whether weight gain can improve PD HRQL will be an important area for future research.

Strengths of the current study included the use of a large national outcomes database drawn from Centers of Excellence worldwide. Several limitations were encountered in this study and may have biased the results. The use of only experienced PD centers may have provided a sampling bias. The database lacked mood (depression) indices, beyond the ‘emotion’ dimension of the PDQ-39. Additionally, the NPF-QII dataset did not collect exact medications and dosages. Furthermore, the calculation of monthly weight change was averaged over the follow-up interval (12±6 months) which tends to minimize monthly fluctuations in weight and potential impact. A more narrow range may have yielded more accurate results but would have reduced the power of the study. Lastly, our study used absolute weight change rather than percent-weight change, which can have differing implications in individuals with varying body weight. It should be noted that direct causation could not be established due to the design of the study. Despite these limitations, the findings were robust and indicate that weight is a potential important factor to consider in the care of the PD patients.

## Conclusion

This large real-world prospective database revealed that multiple factors were associated with interval weight loss in PD. The most unique aspect to the study was the quantification of a longitudinal relationship between weight loss and HRQL. Whether intervention to prevent weight loss can improve PD HRQL could be an important area for future research.
